# A realist inquiry to examine the implementation of Advanced Nurse Practitioner roles in General Practice settings: A realist review protocol

**DOI:** 10.12688/hrbopenres.14319.1

**Published:** 2026-01-15

**Authors:** Pamela McCann, Wayne Thompson, Sean Paul Teeling

**Affiliations:** 1 Nursing, Midwifery and Health Systems, University College Dublin School of Nursing Midwifery and Health Systems, Dublin, Leinster, D04 V1W8, Ireland; 2 UCD Centre for Interdisciplinary Research, Education and Innovation in Health Systems, University College Dublin School of Nursing Midwifery and Health Systems, Dublin, Leinster, D04 V1W8, Ireland; 3 Centre for Person-Centred Practice Research, Queen Margaret University School of Health Sciences, Musselburgh, Scotland, EH21 6UU, UK

**Keywords:** Advanced Nurse Practitioner, Advanced Practice Nurse, General Practice, Family Practice, Primary Care, Realist Review

## Abstract

**Background:**

In Ireland, an ageing population with increasingly complex medical needs and a reduced workforce capacity pose significant challenges for healthcare planning and resourcing. To support capacity building, many countries are seeking innovative workforce solutions to meet the growing demand for healthcare. Expanded nursing roles, such as advanced practice nurses, are being introduced to general practice in many countries. In Ireland, the Advanced Nurse Practitioner (ANP) role is predominant in acute hospitals and integrated care settings. However, an increasing number of nurses working in general practice are pursuing advanced nursing practice. This realist review protocol outlines a framework for evaluating evidence on the implementation of the ANP role in general practice. The review aims to gather robust evidence to generate theories about the implementation of the ANP role and, critically, to understand how the role is implemented, for whom, in what circumstances, in what respects, and how.

**Methods:**

The protocol outlines five critical steps required to conduct a realist review: 1) defining the scope and developing initial theories, 2) developing the search strategy, 3) extracting data and reviewing evidence, 4) synthesising evidence and formulating conclusions and 5) refining theory and disseminating findings. Each stage outlines the specific expectations for that stage of the review.

**Conclusion:**

The implementation of the ANP role in Irish general practice is a new phenomenon and requires further understanding and exploration. It is envisaged that this protocol will establish a robust framework for evaluating the ANP role in general practice. The insight generated by this realist review will, in turn, help determine if the advanced nurse practitioner role has a worthy place in the future of Irish general practice.

## Introduction

In Ireland, an ageing population with increasingly complex medical needs, combined with rising immigration and diminished workforce capacity, presents significant challenges for senior policymakers in healthcare planning and resource allocation ( Collins & Homeniuk, 2021; Connolly & Flanagan, 2024; Connolly *et al.*, 2022). Reflecting this growing and ageing demographic, a substantial increase in demand for general practice services is forecasted in the coming years ( Walsh *et al.*, 2021). To enhance capacity, many countries are exploring innovative workforce solutions to meet the escalating demand for healthcare ( Maier *et al.*, 2022; Scanlon *et al.*, 2023). Expanded nursing roles are being integrated into general practice across numerous nations ( Adams *et al.*, 2024; Hamel *et al.*, 2020; Schlunegger *et al.*, 2023; Wheeler *et al.*, 2022). International systematic literature reviews recognise the role of the Advanced Nurse Practitioner (ANP) in primary care in improving patient access, reducing waiting times and hospital admissions ( Horton *et al.*, 2025; Rossiter *et al.*, 2023; Savard *et al.*, 2025), and increasing patient satisfaction ( Barnett *et al.*, 2022). The ANP role is well established in primary care settings in countries such as Australia, New Zealand, the United Kingdom, and Europe ( Evans *et al.*, 2020; Strachan *et al.*, 2022). Notably, 60% of the nurse practitioner workforce in New Zealand is employed in primary care, working across general practice and public-sector settings ( Mustafa *et al.*, 2021). This contrasts with Ireland, where the ANP workforce predominantly works in the acute hospital environment ( Doherty, 2025). This paper presents a protocol for examining the ANP's role in general practice, understanding it in depth, and assessing whether it warrants inclusion alongside other countries in Irish healthcare.

### The ANP role: General practice context

Traditionally, general practice has been recognised as the primary point of access to healthcare and as a gateway to hospital specialists ( Dunlea *et al.*, 2023). As with acute hospital services, capacity in Irish general practice is under considerable strain, substantially impacting both patients and the GP community ( Irish College of General Practitioners (ICGP), 2023). With an ageing population, a notable increase in demand for general practice services is anticipated in the coming years ( Walsh *et al.*, 2021). A recent preliminary study exploring the views of nurses working in general practice (GPNs) and General Practitioners (GPs) on expanding the nursing role found that 94% of GPs and 81% of GPNs regarded an expanded nursing role as a priority ( Bury *et al.*, 2021). Therefore, there is evident enthusiasm among clinicians to develop the nursing role within general practice.

Regardless of their practice area, ANPs possess competencies within their discipline that enable them to be senior decision-makers, work collaboratively, demonstrate clinical leadership and research skills, conduct advanced physical and mental health assessments, and implement evidence-based interventions, including prescribing, when necessary, for service users with complex and multiple needs ( NMBI, 2025). In general practice, the first registered ANP was appointed in 2005 ( Doherty, 2024). Despite the Department of Health ( DOH, 2019) policy aimed at increasing the number of ANPs to meet emerging and future needs, the development of this role within general practice has been notably delayed. Anecdotal evidence suggests that since the first registered ANP in general practice in 2005, approximately 28 ANPs are now registered, with 25 candidate ANPs currently training ( Loftus-Moran *et al.*, 2024). Most registered ANPs working in general practice are trained within the setting, with only a small number (n = 3) trained in acute care ( Loftus-Moran *et al.*, 2024). The role is described as ‘generalist’, with ANPs in general practice providing autonomous care across various domains, including women’s health, chronic disease management, obesity, and preventive medicine ( Loftus-Moran *et al.*, 2024).

### Rationale: The need to research

Sláintecare, Ireland’s current healthcare reform, is responsible for structured chronic disease programmes in general practice, including prevention programmes and opportunistic screening, and accountable for the recent extension of free GP care to qualifying individuals ( Connolly *et al.*, 2022; HSE, 2023). Crosbie *et al*. (2020) argue that Sláintecare has dramatically increased the workload on a service already strained by reduced workforce capacity and an ageing GP population. Real concerns now exist about the impact of this health reform and the lack of staff to meet increased demand ( Connolly *et al.*, 2022; Crosbie *et al.*, 2020; Doolan & Prior, 2020). Despite international evidence demonstrating the ANP’s positive contribution to workforce capacity ( Horton *et al.*, 2025; Rossiter *et al.*, 2023), the ANP's role in general practice remains unexplored in Ireland, and little is known about its impact on patient outcomes, service efficiency, and the delivery of healthcare policy.

Investigating the effects of the ANP in GP is endorsed by global nursing leaders, who acknowledge that nurses in primary healthcare should be allowed to work to the full scope of their practice ( WHO, 2020). Nationally, the *‘Report of Expert Review Body of Nursing and Midwifery’* ( DOH, 2022) highlights that implementing advanced nursing roles, including those in general practice settings, requires understanding their impact, as well as the barriers and facilitators. The report recognises that research and evaluation in this area are necessary. It is timely, therefore, as the ANP role in GP emerges, to intricately explore, understand, critically analyse, and impart knowledge that will enhance its implementation in Irish general practice settings.

### Use of realist inquiry

Developed in 1997 by Pawson and Tilley, realist inquiry seeks to understand the theories behind social interactions and gain a deeper understanding of *‘what works for whom, in what circumstances, in what respect, and how?’* ( Pawson & Tilley, 1997). This approach is gaining increasing popularity within healthcare, primarily due to the inherent complexity of healthcare systems ( Hunter *et al.*, 2022; Jack, 2022; Teeling *et al.*, 2021). Realist inquiry seeks to understand complex social phenomena, such as the implementation of a new clinical role (e.g., the ANP role) within an existing primary care workforce ( Eaton *et al.*, 2025). The ANP role in general practice is a dynamic, context-driven service innovation ( Adams *et al.*, 2024; Maier *et al.*, 2022; Schlunegger *et al.*, 2023; Wheeler *et al.*, 2022). However, implementing the ANP role may be considered complex for various reasons. In Ireland, there is no formal educational pathway or competency-based framework in place to support career progression for nurses working in general practice ( Connolly & Flanagan, 2024). Equally, there is a scarcity of professional nursing leadership to guide the establishment of the ANP role in general practice ( Loftus-Moran *et al.,* 2024). To further complicate matters, each general practice is an entity with its own way of working. Therefore, the implementation of the role will vary depending on the specific context.

**Figure 1.**
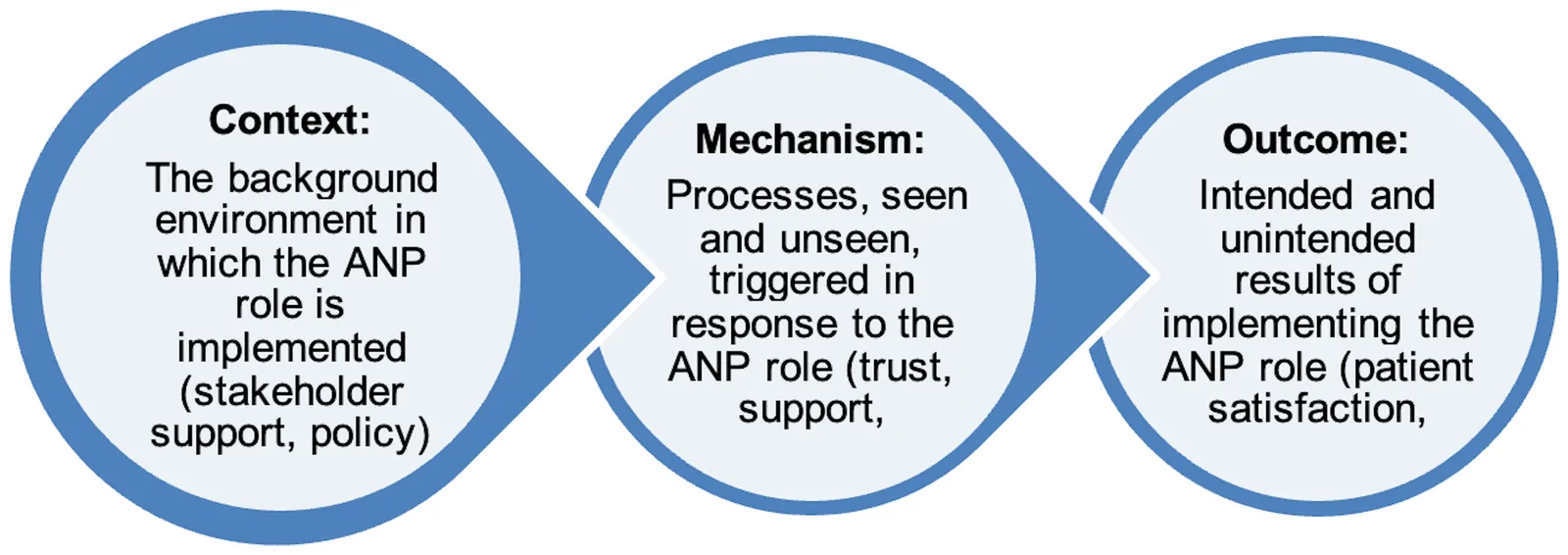
*Study* Context, Mechanism, Outcome Configuration.

Realist inquiry examines how an intervention functions in a particular context. It does this by examining the causal relationships among the principles of context, mechanisms, and outcomes (CMOs) ( Pawson & Tilley, 1997). In realist terminology, this is known as the ‘context, mechanism, outcome configuration’ (CMOc), and is often expressed as *(C) + (M) = (O)* ( Pawson, 2013). A core task of realism is to unearth the processes that may enhance or impede outcomes ( Cooper *et al.*, 2017). Context extends beyond location and may encompass individuals within the programme, as well as interrelationships among stakeholders, the institution, and the wider infrastructure ( Pawson, 2013). Mechanisms are processes that affect the implementation of the ANP role. They are responses and interactions to the intervention and provide a rationale for both intended and unintended outcomes ( Greenhalgh *et al.*, 2017). Outcomes refer to the intended and unintended results of implementing the ANP role, which depend on specific processes or mechanisms that prevail in a particular context. The use of realist inquiry will help elucidate how the complexities of implementing the ANP role in GP interact within specific contexts, producing both intended and unintended outcomes. Thereby understanding what works for whom, in what respects, and why ( Figure 1).

## Protocol

The following protocol outlines a framework for guiding the investigation into the implementation of the ANP role in general practice settings. The following key questions guide this protocol:

*1*  *"What are the mechanisms through which the beliefs and attitudes of key stakeholders about the perceived value or demands of the ANP role in general practice shape the context for its integration into the practice team?"*
*2*  *"In what contexts do organisational and cultural factors act as enablers or barriers to implementing the ANP role in general practice, and what mechanisms within these contexts influence the outcome of successful or unsuccessful role implementation?"*
*3*  *"What are the outcomes of implementing the ANP role in general practice, such as impacts on patient outcomes, service efficiency, and practice performance, and how do specific contexts and mechanisms underpin these outcomes?"*

### Methods

To ensure a robust and credible review, the ‘ *Realist and meta-narrative evidence synthesis: Evolving Standards (RAMESES)'* guidelines provide structure for realist researchers to carry out a review. This protocol follows the key stages recommended by RAMESES. As the realist review will be iterative, it is essential to note that these stages will overlap, with the researcher revisiting and refining each stage as needed. The stages are as follows:

1. Define the scope of the review and develop initial theories
2. Develop the search strategy
3. Data extraction and review of evidence
4. Synthesise evidence and develop conclusions
5. Refine theory iteratively and disseminate findings

***1. Define the scope of the review and develop initial theories***

Determining the scope of the review and developing initial programme theories will be a collaborative effort between key stakeholders and experts within the field. Critical to the initial steps in developing theory will be establishing a local reference group (LRG). During group consultation, initial hunches regarding the ANP role will be discussed. Hunches highlight plausible causal pathways and areas of potential relevance ( Pawson & Tilley, 1997). Following the meeting, a high-level literature review will be conducted. This targeted search aims to map the evidence supporting the initial ideas, thereby ensuring that theories align with broader evidence ( Fawkes & Ward, 2022). Preliminary theories, known as Candidate Programme Theories (CPTs), are initial hypotheses that propose how implementation of the ANP role in general practice might function under specific circumstances ( Wong *et al.*, 2013). Each CPT will be developed utilising the CMO configuration. By combining LRG insights with relevant literature, rough theories will begin to emerge, forming CPTs. The formation of CPTs is, therefore, a strategic process of identifying and locating evidence that aligns with the initial hypotheses and the intervention's contextual understanding. This iterative, purposeful approach enables the development of theories closely aligned with the ANP’s general practice-specific aims and challenges. These CPTs are theories or hypotheses based on the understanding that the implementation of the GP ANP role works/does not work because the actions of underlying mechanisms are activated only in particular contexts ( Pawson, 2013).

***2. Develop the search strategy***

Once CPTs have been formalised in conjunction with the LRG and key literature, they will be presented to an expert panel for adjudication. An expert panel comprises members external to the research team who bring a range of expertise to the research topic, including lived experience, professional knowledge, policy expertise, and academic knowledge ( Power *et al.*, 2023). The expert panel will play a key role in overseeing the research process and will provide valuable perspective and insight at various stages of the review ( Power *et al.*, 2023). Once CPTs have been expertly adjudicated, they are referred to as Initial Programme Theories (IPTs). At this stage, a comprehensive scoping review will be conducted. Searching in a realist review is guided by the review's objectives and focus and is revised iteratively as data emerge ( Wong *et al.*, 2014). The search strategy will utilise a librarian's skills and involve multiple searches of medical and nursing electronic databases, including Medline, PubMed, CINAHL, EMBASE, and EBSCO. Following RAMESES guidance for realist reviews ( Wong *et al.*, 2013), the search strategy will be developed and refined iteratively as programme theories are adjudicated and tested. Searching in realist reviews is therefore theory-driven and adaptive, with search terms refined as understanding of relevant contexts and mechanisms develops, rather than fixed at the outset. This approach is consistent with peer-reviewed realist review protocols ( Keown *et al.*, 2024; Omisore *et al.*, 2025). Table 1 below outlines the inclusion and exclusion criteria, based on the PICO framework, that will maintain the focus of the search strategy ( Schardt *et al.*, 2007). This framework represents the population under study, the intervention, and the outcome.

**Table 1.** PICO framework to identify inclusion and exclusion criteria.

PICO Element	Key Concept	Inclusion and Exclusion Criteria
Population	- General practice settings that employ a candidate or registered ANP - Stakeholders* who have experience working with/ or receiving care from an ANP in General Practice (*GPs, GP Registrars, Practice managers, ANPs, General Practice Nurses, Phlebotomists, Health Care Assistants, Physician Associates, Pharmacists, Administration staff, Service users)	**Included:** - All GP settings, inclusive of single/ group practices, primary care centres and corporately owned GP practices throughout the northern and southern hemisphere that employ an ANP, or equivalent --ANPs that transition from other disciplines (e.g. Emergency Medicine) to work in GP settings (or equivalent settings) - ANPs that work in independent practice in general practice settings - Candidate ANPs working in general practice who are currently undergoing academic/ clinical training to become advanced nurse practitioners’ **Excluded:** - General practice settings that do not employ a candidate or registered ANP in Ireland and internationally, and therefore have no experience working with or receiving care from an ANP in general practice
Interventions	Implementation of the ANP role in general practice	**Included**: Studies that report on the implementation of ANPs and the outcomes identified below
Context	Time Language Setting	- Studies published from 1960 to the present will be considered to capture the international emergence and evolution of advanced practice nursing roles relevant to general practice -English - General Practice/ Family Practice/ Primary Care or equivalent setting - Mixture of both publicly funded and privately owned settings
Outcomes	- Service delivery - Stakeholder perspective - Development of role - ANP perspective	**Included**: - Studies that report on the ANP working in general practice in terms of enhancing or challenging service delivery in general practice, for example, access to care, patient diagnosis and treatment - Studies that report on patients’ subjective experience of engagement with ANP-led care in general practice settings - Studies that report on stakeholder beliefs and attitudes of the ANP role in general practice - Studies that report on factors that may influence how the ANP's role in general practice grows and expands, for example, education and leadership - Studies that report on how ANPs in general practice perceive their role in terms of personal characteristics - Studies that report on ANPs' perception of their role in terms of the transformation and sustainability of general practice **Excluded Studies:** - Studies that do not report on the required outcomes

***3. Data extraction and review of evidence***

Data from the scoping review will be imported into Covidence. Covidence is a platform that streamlines systematic reviews, enabling reviewers to conduct them efficiently through title and abstract screening, full-text screening, data abstraction, and quality assessment ( Hupe, 2025). Imported data will be scrutinised in line with the Preferred Reporting Items for Systematic Reviews and Meta-Analysis (PRISMA) guidelines. Two reviewers (PMcC and WT) will screen data based on title and abstract, followed by an eligibility assessment based on a full article review. A third reviewer (SPT) will adjudicate any discrepancy that may arise in the screening and eligibility process. A realist review draws on a wide range of sources, including grey literature and commentaries ( Jagosh, 2019). While assessing the literature for eligibility in the review, data will be evaluated for relevance, richness, and rigour ( Dada *et al.*, 2023). Relevance determines whether the data can contribute to theory building, and rigour assesses whether the method used to generate the data is credible and trustworthy ( Dada *et al.*, 2023). Richness implies evaluating data sources for both ‘conceptual richness and thickness’ ( Booth *et al.*, 2013).

***4. Synthesise evidence and develop conclusions***

**Figure 2.**
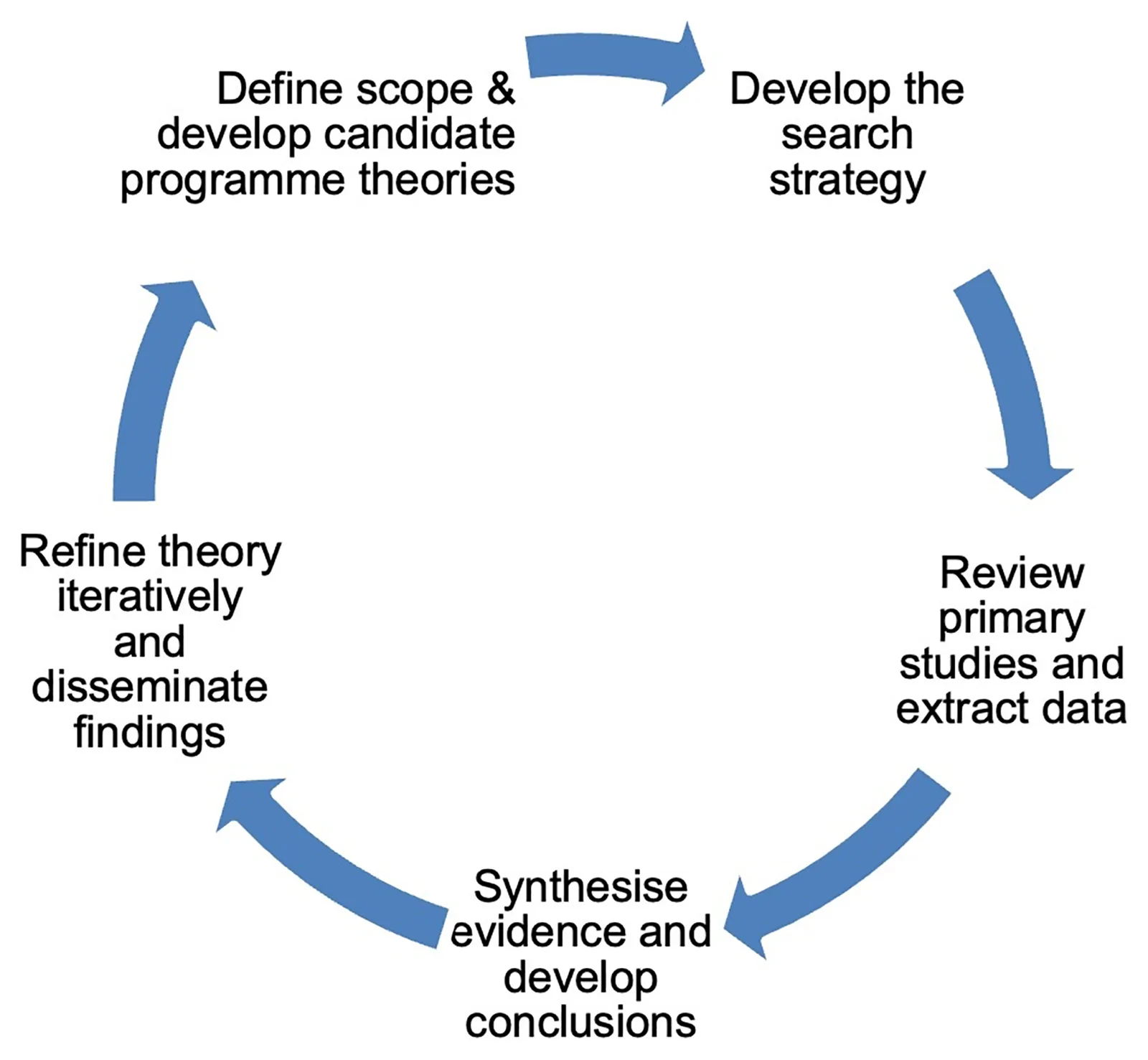
Iterative stages of Realist Review. Adapted from Pawson and Tilley (1997) and RAMESES guidance ( Wong *et al.*, 2013), illustrating the iterative development and refinement of programme theory in realist review.

Once the data has been extracted and reviewed, the eligible literature will be analysed to develop theories and conclusions. To assist with evidence synthesis, a specialised qualitative data analysis software, NVivo, will be utilised. NVivo has features that help to code key concepts, identify C-M-O configurations, and recognise patterns in data ( Bergeron & Gaboury, 2020; Omisore *et al.*, 2025). Whilst synthesising evidence, a key tenet is to understand causation and how causal mechanisms are shaped and constrained by social context, thereby influencing the impact of the ANP role ( Wong *et al.*, 2014). To understand causation and the nature of mechanisms, realists employ ‘realist logic’ and analytic techniques to analyse their data ( Duddy & Wong, 2023; Wong *et al.*, 2013). Using the activities of retroduction and abduction, theories guide the extraction of knowledge about the ANP's role in GP from the literature, to uncover generative causation. Retroduction employs a non-linear process, sifting back and forth between programme theories developed and tested in the review, and the evidence included in the review ( Dada *et al.*, 2023; Jack, 2022). Once evidence is synthesised and conclusions drawn about the implementation of the ANP role in general practice, findings will be further refined before dissemination. Figure 2 provides an overview of the iterative stages and the constant refinement of theory across the review.

***5. Refine theory iteratively and disseminate findings***

Final refinement will occur in consultation with the Expert Panel. Robust theories informed by the review will be presented to the expert panel for refinement and further consideration and will become known as ‘programme theory’. When disseminating the outcomes of this realist review, the reviewers will adhere to the RAMESES reporting guidelines. Outcomes will be published in peer-reviewed journals and presented at conferences to interested audiences, both within academia and the healthcare sector. Furthermore, the outcomes of this review will inform the next phase of realist inquiry, specifically realist evaluation, where the reviewers test the outcomes in a real-world setting. It is anticipated that this realist review and the subsequent realist evaluation will culminate in the award of a PhD, thereby contributing to and expanding the body of scientific knowledge.

## Conclusion

To conclude, this protocol outlines the key stages of a proposed realist review, which aims to examine the existing literature on the implementation of the ANP role in GP. This protocol outlines the step-by-step process for this review and acknowledges that it will be iterative, with stages overlapping. The protocol recognises that a realist review is a complex process and will require the use of recognised software, as well as effective communication between the reviewers and the expert panel. This protocol outlines a concise framework for investigating the implementation of the ANP role in Irish general practice settings, with the goal of understanding specifically what works for whom *,* in what circumstances, in what respects, and importantly, how.

## Ethics

Ethical approval is not required for this protocol, as it involves a review or synthesis of existing literature and no primary data collection.

## Systematic review registration

This protocol has been registered in the PROSPERO database for systematic review and is currently awaiting a registration number, which will be added once available.

## Reporting guidelines

The Realist Review will be informed by the RAMESES (Realist and Meta-narrative Evidence Synthesis: Evolving Standards) project.

## Data Availability

No data are associated with this article Figshare: PRISMA-P 2015 Checklist: A realist inquiry to examine the implementation of Advanced Nurse Practitioner roles in General Practice settings: A realist review protocol. https://doi.org/10.6084/m9.figshare.30880178.v1 ( McCann *et al.*, 2025). Data are available under the terms of the Creative Commons Attribution 4.0 International license (CC-BY 4.0).
