# Supplementary material for: A realist inquiry to examine the implementation of Advanced Nurse Practitioner roles in General Practice settings: A realist review protocol
Source: HRB Open Res. 2026 Jul 7;9:9. Originally published 2026 Jan 15. [Version 2] doi: 10.12688/hrbopenres.14319.2 (PMC13560641; doi:10.12688/hrbopenres.14319.2)
Supplement: Peer review history

# Reviewer report: Reviewer response for version 2

**DOI:** 10.21956/hrbopenres.15943.r57008

**Published:** 2026-08-06

**Authors:** Suryanti Chan

**Affiliations:** 1 Universitas Dian Nuswantoro, Semarang, Indonesia

This manuscript presents a protocol for a realist review examining the implementation of Advanced Nurse Practitioner (ANP) roles in General Practice settings. The topic is relevant and timely, and the use of a realist approach is appropriate for examining how, why, for whom, and under what contextual circumstances ANP roles are successfully implemented.

 Overall, the Methods section is well structured and demonstrates appropriate alignment with RAMESES guidance. The authors provide a clear description of the iterative stages of the realist review, including the development and refinement of Candidate and Initial Programme Theories, stakeholder and expert involvement, literature searching, study selection, data extraction, assessment of relevance, richness and rigour, and theory-driven synthesis using CMO configurations, retroduction, and abduction. The use of two reviewers for screening and data extraction, with a third reviewer to resolve disagreements, further strengthens the methodological approach.

 I rated the reproducibility of the methods as “Partly”, primarily because some procedural details could be reported more explicitly. Although iterative and theory-driven searching is an inherent and appropriate feature of realist reviews, the protocol would benefit from providing at least one complete illustrative search strategy for a key database (for example, MEDLINE), including the full search syntax, Boolean operators, field specifications, and any limits applied. This would improve transparency regarding the initial search while still allowing subsequent searches to evolve iteratively in response to programme theory development.

 The authors should also clarify how subsequent iterative searches and modifications to the search strategy will be documented and reported. Maintaining an audit trail of additional searches, search terms, dates, sources searched, and the theoretical rationale for each iteration would considerably strengthen the transparency and reproducibility of the review process.

 In addition, the manuscript states that bespoke data extraction templates will be developed for each Initial Programme Theory. Providing an example of the proposed extraction template, or including it as supplementary material, would help readers understand how evidence relating to contexts, mechanisms, outcomes, relevance, richness, and rigour will be consistently identified and recorded across reviewers.

 These comments should not be interpreted as requiring the realist review to be reproducible in exactly the same manner as a conventional systematic review. The iterative and interpretive nature of realist synthesis is acknowledged and appropriate. Rather, the suggested additions would provide a clearer audit trail and enhance methodological transparency, allowing readers to understand and, as far as reasonably possible, follow the analytic pathway from evidence identification to programme theory refinement.

 Overall, I consider the protocol methodologically appropriate and potentially valuable. The main revisions required relate to improving transparency and documentation of the search and data extraction procedures rather than changing the overall realist review methodology.

Is the study design appropriate for the research question?

Yes

Is the rationale for, and objectives of, the study clearly described?

Yes

Are sufficient details of the methods provided to allow replication by others?

Partly

Are the datasets clearly presented in a useable and accessible format?

Not applicable

Reviewer Expertise:

Health promotion, healthcare quality assurance, public health, medical education

I confirm that I have read this submission and believe that I have an appropriate level of expertise to confirm that it is of an acceptable scientific standard, however I have significant reservations, as outlined above.

# Reviewer report: Reviewer response for version 1

**DOI:** 10.21956/hrbopenres.15762.r53180

**Published:** 2026-04-03

**Authors:** Monica O'Reilly-Jacob, Sarah Giordano

**Affiliations:** 1 Columbia University School of Nursing, New York, New York, USA; 2 Nursing, Columbia University School of Nursing (Ringgold ID: 15760), New York, New York, USA

The protocol tackles an important and timely implementation problem and, overall, it has a strong conceptual fit with realist inquiry: the authors clearly position ANP-in-general-practice as a complex, context-dependent service innovation and appropriately anchor their approach in RAMESES-guided realist review stages and CMO thinking.

 The stepwise structure (scope/theory building → searching → extraction/appraisal → synthesis → iterative refinement) and the planned engagement of a local reference group and expert panel are real strengths that should support practice-relevant theory development and keep the review grounded in stakeholder realities.

 To make the protocol more scientifically robust and replicable, the main opportunity is to operationalize several key realist steps with more procedural specificity:

 (1) tighten the objectives by naming a small set of candidate **programme theory domains they plan to test/refine (e.g., regulation/scope, leadership, role clarity, team acceptance), so the review remains focused rather than overly broad;

 (2) add reproducible search detail (at least one full example search string per database, grey literature approach, citation chasing/cluster searching plan, and stopping rules); (3) provide a concrete CMO-oriented extraction/coding template (what counts as “context,” “mechanism,” “outcome,” who codes, how disagreements are handled) and specify how they will move from codes → CMOCs → refined programme theory;

 (4) define how “relevance/richness/rigour” will be judged in practice (criteria and workflow), and explicitly note whether/how they will triangulate across study types or data sources to strengthen causal inference; and

 (5) consider feasibility by narrowing scope (e.g., prioritizing one or two outcomes, limiting to certain settings, consider limiting to non-US studies as the implementation of APN roles in the US 60 years ago started from zero where Ireland has the ability to learn from models currently in practice, or focusing on qualitative/process evidence first).

 With these additions, the protocol would retain its clear rationale and strong realist alignment while becoming much more transparent, feasible, and reproducible. Overall, a promising and well-motivated piece of work that should make a valuable contribution once these clarifications are incorporated.

Is the study design appropriate for the research question?

Partly

Is the rationale for, and objectives of, the study clearly described?

Partly

Are sufficient details of the methods provided to allow replication by others?

Partly

Are the datasets clearly presented in a useable and accessible format?

Not applicable

Reviewer Expertise:

health services research, nurse practitioner workforce, primary care, US-based

We confirm that we have read this submission and believe that we have an appropriate level of expertise to confirm that it is of an acceptable scientific standard, however we have significant reservations, as outlined above.

# Response

**Published:** 2026-05-12

**Authors:** Sean Paul Teeling

Dear Dr O’Reilly-Jacob and Dr Giordano

 Thank you sincerely for your thoughtful and constructive review of our protocol. We greatly appreciate your recognition of the conceptual alignment between the proposed review and realist inquiry methodology, as well as your acknowledgement of the value of the staged realist approach and stakeholder engagement processes incorporated within the protocol.

 We are particularly grateful for your recommendations regarding the need for greater procedural specificity and methodological clarity. In response to your comments, we have made several amendments throughout the manuscript to strengthen the scientific robustness, transparency, and reproducibility of the protocol.

 First, Stage 1: Define the Scope of the Review and Develop Initial Theories has been amended (lines 151–156) to further clarify the candidate programme theory domains that will initially be explored within the review. These now include leadership, communication and collaboration, education, and research as key domains of advanced practice, thereby supporting a more focused and theoretically informed review process.

 Second, to strengthen the reproducibility of the search process, Stage 2: Develop the Search Strategy has been revised to include a table outlining the keywords used across databases (see line 182). Additional detail has also been included regarding the iterative nature of realist searching and evidence identification.

 Third, Stage 3: Data Extraction and Review of Evidence has been expanded to provide greater procedural detail regarding the CMO-oriented extraction process and reviewer procedures (lines 210–219). This now clarifies that bespoke extraction templates will be used to identify contextual factors, mechanisms, and outcomes relevant to each IPT, that two reviewers (PMcC and WT) will independently undertake data extraction, and that SPT will assist in resolving discrepancies where required. Additional text has also been included to further describe how extracted data will support the refinement of CMOCs and programme theories throughout the review process.

 In response to your recommendation regarding relevance, richness, and rigour, additional clarification has been inserted at lines 229–245 outlining how these concepts will be operationalised and assessed during evidence appraisal, including consideration of the contribution different evidence sources may make to programme theory development and refinement.

 Finally, in response to your comments regarding feasibility and scope, we have added additional text at lines 285–292 to clarify how the review will remain both focused and manageable within a realist methodology framework. Specifically, we now acknowledge the contextual differences influencing ANP role implementation across international healthcare systems and general practice settings, including differing regulatory, organisational, and workforce structures. We further clarify that iterative theory refinement will be used to prioritise evidence most relevant to the programme theories under investigation and the review objectives, thereby supporting a focused and feasible review scope. We believe this addition helps balance contextual learning across healthcare systems with maintaining conceptual clarity and methodological feasibility.

 Please note that, due to tracked changes within the manuscript, line numbers may vary slightly depending on whether the document is viewed in Simple Markup or All Markup mode; however, the referenced sections are correct within the fully marked-up version.

 Thank you again for your valuable engagement with our work and for your constructive recommendations, which have helped strengthen the protocol considerably.

 Kind regards

 Pamela McCann

 Wayne Thompson

 Seán Paul Teeling

# Reviewer report: Reviewer response for version 1

**DOI:** 10.21956/hrbopenres.15762.r54316

**Published:** 2026-03-28

**Authors:** Geraldine Lee

**Affiliations:** 1 University College Cork, Cork, County Cork, Ireland

Overall, a timely examination of ANPs in primary care within the Irish context. A realist review is appropriate and methodology is described in detail. 

 I would recommend the authors consider adding 'Advanced Clinical Practitioner' to their search as ANP is not a title used in the UK (It is not a separate registrable title), otherwise the ACP role in primary care will not be captured in their search and there is a lot of work going on in this area.

 Also, the authors should consider examining 'Hospital in the Home' as although it is not primary care in the traditional sense, it is providing ANP level care in a primary care setting.

 With the scoping: local reference group- need some detail on who this group consists of and how they will be identified.

 Further details needed too on the expert panel. The protocol states that it will include ‘lived experience, professional knowledge, policy expertise, and academic knowledge.’ But it is not clear if this is referring to patients, healthcare professionals, etc.

 Greater clarity on the membership of the expert panel is required.

Is the study design appropriate for the research question?

Yes

Is the rationale for, and objectives of, the study clearly described?

Yes

Are sufficient details of the methods provided to allow replication by others?

Yes

Are the datasets clearly presented in a useable and accessible format?

Not applicable

Reviewer Expertise:

Advanced practice, nursing regulation, health services research

I confirm that I have read this submission and believe that I have an appropriate level of expertise to confirm that it is of an acceptable scientific standard.

# Response

**Published:** 2026-05-12

**Authors:** Sean Paul Teeling

Dear Professor Lee

 Thank you sincerely for your thoughtful and constructive feedback on our protocol. We greatly appreciate the time and expertise you have contributed to reviewing the manuscript.

 In relation to your suggestion regarding the inclusion of the term “Advanced Clinical Practitioner” (ACP), we recognise the importance of this role within the UK context, particularly in primary care settings. However, for the purposes of this realist review, we intentionally limited the scope to Advanced Nurse Practitioner (ANP) roles as defined within our PICO framework. While we acknowledge there may be overlap between ACP and ANP roles, we considered that including both terms could broaden the review beyond its intended nursing-specific focus and potentially reduce conceptual clarity during programme theory development. We have now clarified this rationale within the manuscript.

 Similarly, we appreciate your suggestion regarding “Hospital in the Home” services. While these services may involve ANPs delivering advanced-level care within community settings, we considered them to represent a distinct care context from first-contact general practice, which is the primary focus of this review. We were mindful that including these services could introduce additional contextual variation that might limit the development of clear and transferable programme theories specific to ANP roles in general practice. This rationale has also now been clarified within the protocol.

 In response to your comments regarding stakeholder involvement, we have strengthened the protocol to provide additional detail on both the local reference group and the Expert Panel. Specifically, Stage 1 now includes further clarification regarding the composition and identification of the local reference group (lines 133–138), while Stage 2 provides greater detail regarding the membership and expertise represented within the Expert Panel, including lived experience, clinical, policy, and academic perspectives (lines 163–175).

 Please note that, due to tracked changes within the document, line numbers may vary slightly depending on whether the manuscript is viewed in Simple Markup or All Markup mode; however, the referenced sections are correct within the fully marked-up version.

 Thank you again for your valuable comments and engagement with our work.

 Best wishes

 Pamela McCann

 Wayne Thompson

 Seán Paul Teeling
